# Machine learning deciphers structural features of RNA duplexes measured with solution X-ray scattering

**DOI:** 10.1107/S2052252520008830

**Published:** 2020-08-12

**Authors:** Yen-Lin Chen, Lois Pollack

**Affiliations:** a School of Applied and Engineering Physics, Cornell University, Ithaca, New York 14853, United States

**Keywords:** ribonucleic acids, machine learning, solution X-ray scattering, wide-angle X-ray scattering, computational modelling

## Abstract

Macromolecular structures can be determined from solution X-ray scattering. Small-angle X-ray scattering (SAXS) provides global structural information on length scales of 10s to 100s of Ångstroms, and many algorithms are available to convert SAXS data into low-resolution structural envelopes. Extension of measurements to wider scattering angles (WAXS or wide-angle X-ray scattering) can sharpen the resolution to below 10 Å, filling in structural details that can be critical for biological function. These WAXS profiles are especially challenging to interpret because of the significant contribution of solvent in addition to solute on these smaller length scales. Based on training with molecular dynamics generated models, the application of extreme gradient boosting (XGBoost) is discussed, which is a supervised machine learning (ML) approach to interpret features in solution scattering profiles. These ML methods are applied to predict key structural parameters of double-stranded ribonucleic acid (dsRNA) duplexes. Duplex conformations vary with salt and sequence and directly impact the foldability of functional RNA molecules. The strong structural periodicities in these duplexes yield scattering profiles with rich sets of features at intermediate-to-wide scattering angles. In the ML models, these profiles are treated as 1D images or features. These ML models identify specific scattering angles, or regions of scattering angles, which correspond with and successfully predict distinct structural parameters. Thus, this work demonstrates that ML strategies can integrate theoretical molecular models with experimental solution scattering data, providing a new framework for extracting highly relevant structural information from solution experiments on biological macromolecules.

## Abstract 2

A supervised machine learning approach is applied to integrate molecular dynamics simulations with solution X-ray scattering of double-stranded ribonucleic acid (dsRNA) structures with a focus on extracting critical structural information hidden in the wide-angle regime.

## 1. Introduction   

Ribonucleic acids (RNAs) comprise an important class of biological macromolecules that not only transfer genetic codes, but also signal their response to binding partners through structural changes. Since the discovery of the first catalytically active RNA in the 1980s (Cech *et al.*, 1981 ▸), much attention has been focused on how RNA sequence and structure enable its responses to partners, including ions, small ligands and proteins. For example, flexible and/or single-stranded regions are known to facilitate various conformational states of RNA molecules (Denny *et al.*, 2018 ▸). Recently, two independent studies focused on the structural variation of fully base-paired RNA duplexes, which exhibit sequence-dependent (Yesselman *et al.*, 2019 ▸) and salt-dependent (Chen & Pollack, 2019 ▸) conformations. These works suggest that subtle variations in the RNA stems can affect the precise alignment of contacts that stabilizes tertiary structures, imparting more selectivity to interactions and expanding the biological functionality of RNA (Chen *et al.*, 2018 ▸). Here, we report a new approach for detecting the small twisting and compression of the RNA duplexes which may dramatically impact the overall molecular structure.

Small-angle X-ray scattering (SAXS) has been widely applied to reveal the conformations of biological macromolecules *in vitro*. SAXS maps orientationally averaged macromolecular electron density distributions, measured *in vitro*, to produce a 1D curve by Fourier transform. In SAXS experiments, global structural parameters are readily extracted, ranging from the molecular radius of gyration via Guinier analysis to global structural envelopes (Blanchet & Svergun, 2013 ▸). Established methods provide protocols for modelling structures and solvent shells (Franke *et al.*, 2017 ▸), and even enable the determination of structural ensembles that fit the data when coupled with atomic models and computational algorithms (Mylonas *et al.*, 2007 ▸; Tria *et al.*, 2015 ▸; Shevchuk & Hub, 2017 ▸). With recently developed algorithms, it is now possible to compute low-resolution electron densities for single- or multi-component systems (Grant, 2018 ▸).

Higher spatial resolution can be achieved by acquiring data at larger/wider scattering angles. Wide-angle X-ray scattering or WAXS has been implemented in a few cases to study the effects of crowding on protein conformation (Makowski *et al.*, 2008 ▸), or to determine ensembles of protein (Chen & Hub, 2014 ▸) or nucleic acid (Pabit *et al.*, 2016 ▸) conformations in solution samples. The inclusion of WAXS data, where higher resolution details are mapped, has the potential to enhance the extraction of finer molecular structures from a 1D dataset. WAXS data are much more challenging to interpret than SAXS data. No single global parameter, like radius of gyration, exists to constrain the structures, and WAXS profiles reflect solvent molecules and excluded volumes as well as the numerous small conformational variations *in vitro* (Park *et al.*, 2009 ▸; Nguyen *et al.*, 2014 ▸). Only a few computational tools are available to analyse WAXS data directly (Bardhan *et al.*, 2009 ▸; Knight & Hub, 2015 ▸); thus, the analysis is *ad hoc* and relies on fitting the data with atomic models.

Although challenging to perform and interpret, WAXS experiments can be especially insightful for certain classes of structures. Periodic molecular features are well captured by WAXS, including, for DNA and RNA duplexes, their diameters or the distance between the two phosphate-heavy backbone strands. These periodic structures are manifested as peaks and troughs in 1D scattering profiles (Chen & Pollack, 2019 ▸). Their presence or absence may lead to interpretable changes in WAXS profiles (Tiede *et al.*, 2002 ▸; Zuo *et al.*, 2006 ▸). However, it is a challenge to correlate WAXS fingerprints with specific periodic structures because for nucleic acids many of the structural features, such as helical radius and major groove width, share similar length scales. As a result, without sufficient knowledge of specific macromolecular systems, WAXS data interpretation can be extremely hard to establish.

We recently proposed a new approach for interpreting subtle, salt-induced changes in RNA duplex structure which relies on comparing measured with computed WAXS scattering profiles. Atomically detailed molecular conformations were generated through molecular dynamics (MD) simulations; each structure was subsequently populated with solvent and ions. An ensemble optimization method was employed to select sets of structures whose summed, computed scattering profiles best recapitulate the measurement. We found that only a small number of conformations was required to fit the data and interesting salt-dependent conformational differences were recorded (Chen & Pollack, 2019 ▸).

Despite the broad range of information potentially available, it is important to recognize the intrinsic limited information content of these types of data (Moore, 1980 ▸; Rambo & Tainer, 2013 ▸). The amount of information contained in the SAXS data is determined by the structure of the macromolecule itself; it is not uniformly distributed across the scattering angles (Spill & Nilges, 2017 ▸). While the inclusion of WAXS data increases the information content of solution X-ray scattering, it also introduces unknowns. As inferred above, certain expanses of scattering angles might contain more information on the structures of the molecule than others. These information-rich regions rely, of course, on the specific structural features of interest.

The goal of this work is to describe a new approach for extracting information from and interpreting features of SAXS/WAXS (collectively SWAXS) data using machine learning (ML). ML has been successfully applied to a classification of diverse molecular shapes using SAXS (Franke *et al.*, 2018 ▸). The deep learning model, a special case of ML, was employed for model reconstruction from experimental SAXS profiles via an auto-encoder and decoder network (He *et al.*, 2020 ▸). Here, we focus on the wide-angle regime and demonstrate one way that ML models can aid WAXS data interpretation, specifically when unique structural fingerprints are present on the 10 Å length scale. SWAXS profiles, accurately computed from molecular models with specified and known structural descriptors, can serve as training, validation and testing sets for supervised ML models which, ultimately, are able to ‘recognize’ experimental scattering patterns of unknown conformations.

Due to the complexity of mapping structures onto 1D solution X-ray scattering profiles, simple approaches such as linear regressive models are not suitable for this work. We discuss the application of an ML approach to analyse and interpret SWAXS profiles: extreme gradient boosting (XGBoost) (Chen & Guestrin, 2016 ▸). Full scattering profiles of MD-generated, double-stranded RNA duplex structures are computed and associated with structural descriptors extracted from the molecular models themselves. Features of interest include helical radius, twist, rise, major groove width and A-form fraction. The trained ML models are subsequently applied to predict the corresponding structural descriptors from noisy SWAXS data, either generated from distinct models or experimentally acquired. In this work, we achieved overall high accuracies (low errors) on all the structural descriptors. We also found good agreement of XGBoost models with results from a recent study that used a curve-fitting modelling method to extract structural descriptors. In practice, XGBoost performs well on these data with little effort on hyperparameter tuning and good interpretability.

Our ML approaches bridge theoretical molecular models and experimental data with consideration of errors and transform many *ad hoc* analyses and curve-fitting strategies into a more general framework for different biological systems. The use of raw and full spectra of SWAXS profiles without customized transformation supports the success of information extraction from momentum (*q*) space data by XGBoost.

## 2. Material and methods   

### 2.1. RNA sample and SWAXS measurements   

A single-stranded 12-mer of RNA with sequence 5′-CCU CCU AAU CGC-3′ was purchased from IDT (CoralVille, IA) and annealed with its complement to create a 12-base-paired duplex. Excess and unannealed single strands were separated by spinning the samples in Amicon 3 kDa 0.5 ml Ultra centrifugal filters (EMD Millipore, Billerica, MA) and the samples were subsequently buffer-exchanged to contain 30, 50, 100, 200 or 500 m*M* KCl, and 0.25, 0.50, 1.00 or 5.00 m*M* MgCl_2_. Each buffer contains a background of 10 m*M* K-MOPS and 20 µ*M* EDTA. The divalent ion-containing buffers have an additional 100 m*M* KCl background. The final sample (duplex) concentrations ranged from 130 to 300 µ*M*.

Small- and wide-angle X-ray scattering experiments were conducted at Cornell High Energy Synchrotron Source (CHESS) beamline G1 using two PILATUS 100 K detectors (Dectris AG, Switzerland) with sample-to-detector distances of 1.7 and 0.4 m for SAXS and WAXS data collection, respectively. The scalar value of X-ray photon momentum transfer *q* is defined as 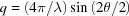, where λ and 2θ are the X-ray wavelength and scattering angle, respectively. The coupled SAXS and WAXS measurements span a *q* range from 0.01 to 0.97 Å^−1^. Absolute calibration was implemented using the molecular weight of our dsRNA system under known conditions.

### 2.2. Dataset preparation   

Approximately 5000 *ab initio* dsRNA duplex conformations were obtained from MD simulations (Templeton & Elber, 2018 ▸) of RNA 12-mers, where details of the MD simulations are provided in the supporting material. In the simulations performed on two tethered 12-mers, we selected the structure of the 12-mer that was allowed to sample all conformations (the other one was fixed). We included all the simulation trajectories in our dataset, including those acquired in solutions containing different salt ions. The sequence of the 12-base-paired RNA duplex from the simulation is identical to the one reported here. As described in the work by Chen & Pollack (2019 ▸), we applied a 3D reference interaction site model (3D-RISM) to model the surrounding (excess) solvent molecules and ions (both cations and anions) for calculation of SWAXS profiles. In order to include solvent and ions and ensure accurate computation of the SWAXS profiles with proper buffer subtraction (Chen & Hub, 2014 ▸, 2015 ▸), the edges of the simulation box are more than 10 Å from all RNA atoms. These full structural models are required to build an accurate, large dataset for training, validation and testing of the XGBoost model. This high-throughput computation of SWAXS profiles was implemented in *Julia* (Bezanson *et al.*, 2012 ▸) on Cornell Red Cloud using a 28-core server node with Intel Xeon E5650 (2.7 GHz, Santa Clara, CA). The calculation takes 2 s for each pair of solute and solvent over 191 *q* points, *q* = 0.000–0.950 Å^−1^ with a spacing of 0.005 Å^−1^. To account for different experimental conditions and the effect of different salts on buffer subtraction, 3D-RISM computations were carried out in solutions containing 30, 50, 100, 200 and 500 m*M* KCl and 0.25, 0.50, 1.00 and 5.00 m*M* MgCl_2_. As a result of imperfections in modelling, an additive constant (*c*) is introduced (Chen & Hub, 2014 ▸; Knight & Hub, 2015 ▸; Schneidman-Duhovny *et al.*, 2013 ▸) to compensate for changing salt conditions. For each theoretical SWAXS curve, *I*(*q*), we determine *c* using the following formula:
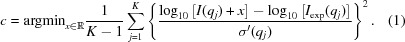
In general, *c* < 10% of the scattering intensity at *q*_max_ = 0.950 Å^−1^. In equation (1), *I*_exp_(*q_j_*) is the absolute calibrated experimental SWAXS intensity. This curve contains data acquired at *K* different *q* values, *q_j_*, and σ′(*q_j_*) is the propagated experimental error:
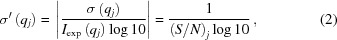
where σ(*q_j_*) and (*S*/*N*)_*j*_ are the experimental error and signal-to-noise ratio at *q_j_*, respectively. Note that equation (1) here is the same as equation (1) in the work by Chen & Pollack (2019 ▸) with the exception of the scaling factor which provides absolute calibration. The input data of our ML models consist of about 50 000 curves representing the logarithms of the corrected SWAXS profiles computed under all solution conditions.

**Figure 1:**
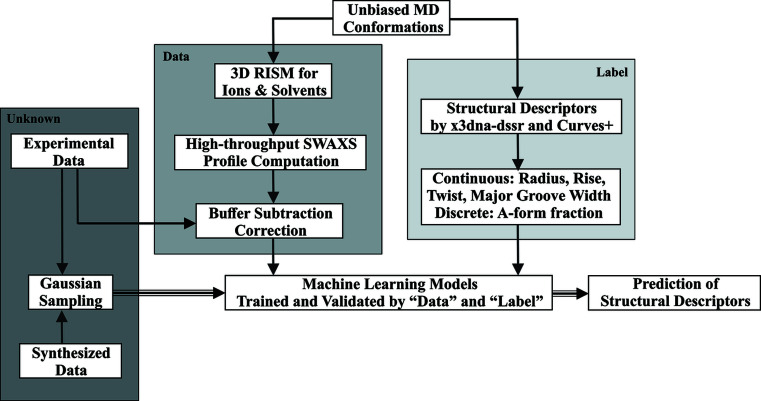
Schematic of the data pipeline. We used structures from unbiased MD simulations to calculate the SWAXS profiles and attached structural descriptors to the profiles using *x3dna-dssr* and *Curves+*. The XGBoost models were trained using 68% of the dataset and the hyperparameters were tuned based on the validation set. The unknown datasets, consisting of one synthesized profile from the testing set and two experimental SWAXS profiles, were sampled and fed into the trained models to predict the corresponding structural descriptors.

The helical parameters of interest are helical radius, twist, rise, A-form fraction and major groove width because they are the determinants of a dsRNA periodic helical structure and are cast as 1D SWAXS features as a result of Fourier transform by X-ray scattering. The structural descriptors of the dsRNA duplexes from the MD simulations are analysed and extracted by the program *x3dna-dssr* (Lu & Olson, 2008 ▸) and used as labels under our supervised learning scheme. The helical A-form fraction is treated as discrete with 12 possible values corresponding to the number of base pairs (0/11, 1/11, … 11/11). Major groove widths were computed by *Curves+* (Blanchet *et al.*, 2011 ▸). The overall data preparation procedure is illustrated in Fig. 1 ▸.

**Figure 2:**
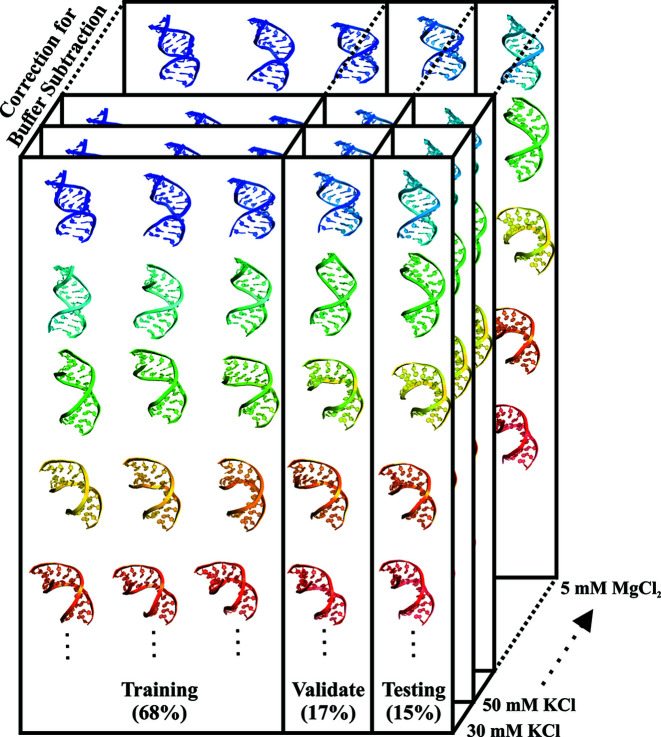
Data-splitting strategy. We split the models into training (68%), validation (17%) and testing (15%) sets based on dsRNA conformations. Each conformation is associated with nine buffer-subtraction-corrected SWAXS profiles that should be kept together.

The full dataset, consisting of about 50 000 computed SWAXS profiles and their associated models, is divided into three parts: training (68%), validation (17%) and testing (15%), as shown in Fig. 2 ▸. The data-splitting strategy was based on random shuffling of the conformations; all the buffer-subtraction-corrected profiles of a specific duplex conformation were included in the same dataset, be it training, validation or testing. This ensured that the SWAXS features of any one specific conformation are contained within the dataset regardless of buffer conditions. The testing set was constructed at the beginning of the ML process and saved as an individual file, unseen by the ML models until the final testing stage. We tuned the hyperparameters of XGBoost, for example, the learning rate and number of training iterations based on the performance on both training and validation sets.

### 2.3. XGBoost   

The extreme gradient boosting (XGBoost) algorithm (Chen & Guestrin, 2016 ▸) is based on classification and regression trees (CARTs) and applies the ensemble idea to construct a linear combination of CARTs with learnable weights (*w_i_*). Suppose an ensemble **T**(*K*) contains *K* CARTs:
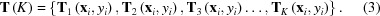
In equation (3), **x** *_i_* is the SWAXS profile with 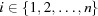 where *n* is the size of training set and *y_i_* is the structural descriptor that characterizes the duplex geometry. Each CART *T_j_* with 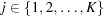 assigns an output 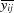 according to the input **x** *_i_*. *T_j_* can be represented as a function *f_j_* which maps the data (scattering profile) to a structural descriptor. The final prediction 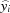 is the sum of all the 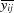 values, *i.e.* the sum of outputs from all CARTs (Chen & Guestrin, 2016 ▸):
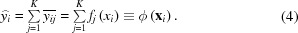
In equation (4) 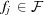, where 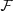 is the functional space of all possible CARTs with specified depth. The training objective is to minimize the regularized loss function defined as follows:
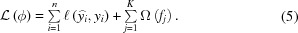
The term 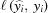 is the loss between the prediction 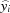 and real label *y_i_* and is chosen to be the mean-squared-error for regression problems and multiclass-cross-entropy for classification problems. The regularization function Ω penalizes the complexity of each CART, *f_j_*, and avoids overfitting. As described in the work by Chen & Guestrin (2016 ▸), Ω contains the penalties on the number of weights, the L1 and L2 norm of the weights, ∣∣**w**∣∣, ∣∣**w**∣∣^2^. The implementation is based on *xgboost* and *scikit-learn* python libraries.

Since this method is based on decision-tree boosting algorithms (Friedman, 2002 ▸; Natekin & Knoll, 2013 ▸), the importance of each feature to the eventual prediction might vary. Two types of importance are of interest in this work. The first is ‘weight-importance’ which reports the number of times a certain feature is used in decision rules. Equivalently, it is the number of times a feature appears at a branching point. Therefore, the weight-importance reveals the decision-making process of the model in predicting a label. The second type of importance is the ‘gain-importance’ which reports the total gain in all outputs 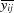 if a certain feature is used in the decision rule. In other words, the ‘gain-importance’ reports the effectiveness of a certain feature in making predictions. In the context of SWAXS profiles, the features are the logarithms of absolute intensities at different *q* values. By reporting both types of importance, we show in a later section that the XGBoost model’s prediction processes are very similar for all the structural descriptors, but intensities at some *q* values appear to influence the prediction more strongly.

### 2.4. Sampling of noisy SWAXS profiles to account for errors   

In modelling solution X-ray scattering data, it is important to report how the experimental errors influence the results (Hub, 2018 ▸). Distinct modelling frameworks propagate the experimental errors differently, and some destabilize the results given small experimental perturbations. These effects might be especially significant in ML models due to their nonlinearity.

Experimentally, the buffer-subtracted solution X-ray scattering intensity can be approximated by a normal distribution according to the central limit theorem. Therefore, the SWAXS intensity, *I*(*q_i_*), can be written as the normal distribution, 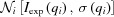, where *I*_exp_(*q_i_*) and σ(*q_i_*) are the experimentally measured intensity and corresponding error at *q_i_*. To simulate data obtained under noisy conditions, we treated each data point as a sampled point from an independent normal distribution, 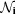, ignoring molecule-dependent covariances between different scattering angles (Spill & Nilges, 2017 ▸). Five thousand SWAXS profiles were sampled from 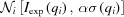 with error level α > 0. We chose low, medium and high error levels corresponding to α = 0.2, 1.0 and 2.0 to investigate how errors affect the final output of our ML models. Note that the signal-to-noise ratio is proportional to α^−1^. The use of multiple curves is essential in obtaining good statistics, especially when sampling from profiles with larger errors frequently yields (unphysical) negative intensities in the high-*q* regime. The 5000 sampled profiles were input into trained ML models to obtain the distribution of structural descriptors.

## 3. Results and discussion   

### 3.1. XGBoost: training, validation and testing results   

**Figure 3:**
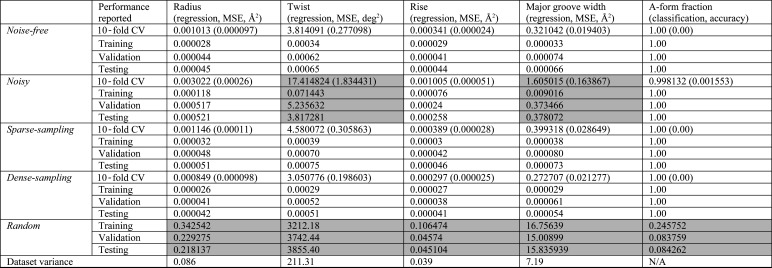
Summary of training, validation and testing of five XGBoost models on different structural descriptors. The variances are reported in the last row. The 10-fold CV results report the averaged regression mean-squared error (MSE) or classification accuracy and the standard deviation among 10 folds. Note that we used 750 and 7500 CARTs in the 10-fold CV and training processes, respectively. The shaded models are identified subjectively as poor, based on 10-fold CV results, performance on all the datasets and comparison with other trained models on the same structural descriptor. Overall, the numbers suggest that the XGBoost model is able to learn or recognize the patterns in the training data and generalize for unknown testing data. This characteristic implies the potential to be applied to noisy experimental data and different molecular systems.

**Figure 4:**
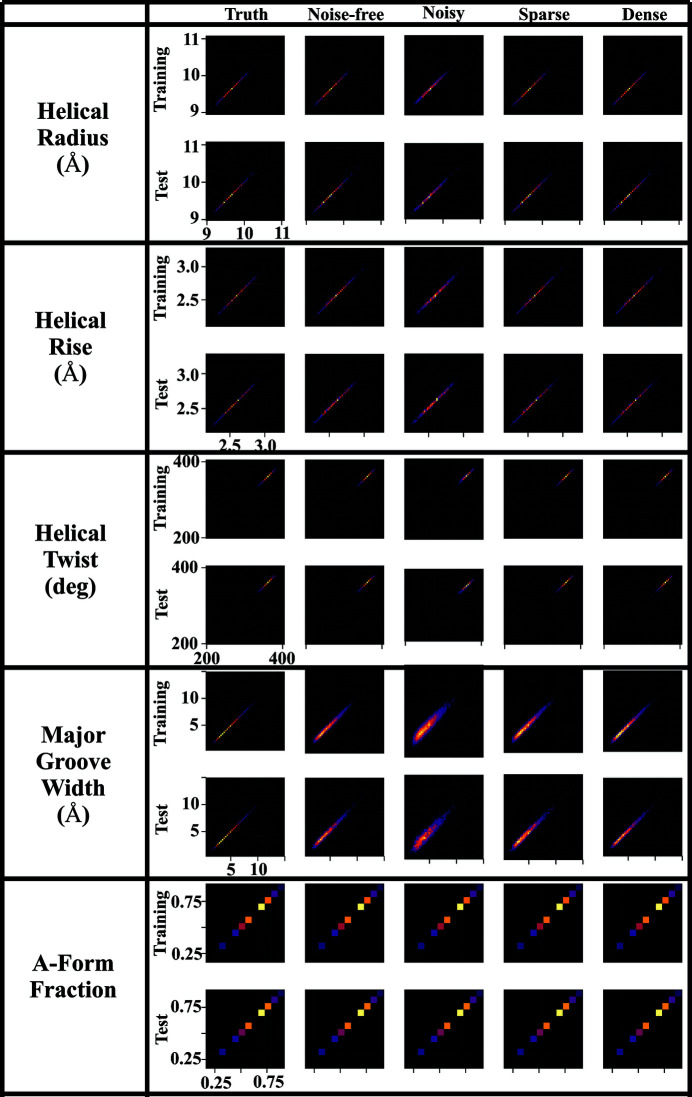
Confusion matrices reporting the performances of all the XGBoost models (*noise-free*, *noisy*, *sparsely sampled*, *densely sampled* in columns 2–5) on different structural descriptors. Compared with the *truth*–*truth* matrices in column 1, all the trained models perform well on both the training set and the testing set, suggesting the ability to generalize for unknown datasets.

To assess the consistency of the ML models and explore the effect of noise on the training data we trained five different XGBoost models for each structural descriptor: *noise-free*, *noisy*, *sparsely sampled*, *densely sampled* and *random*. The *noise-free* model was trained using the aforementioned dataset with 191 *q* points, derived from direct computation based on an MD structure. The *noisy* model was trained using the same training set with 5% Gaussian noise added to the theoretical intensities. Moreover, the theoretical SWAXS profile is smooth, so one can sample an arbitrarily small or large number of intensities from the profiles. We further trained the *sparsely sampled* and *densely sampled* model using 100 and 400 uniformly sampled intensities as reduced and augmented features. Finally, to test the sensitivity of XGBoost to underlying SWAXS patterns and to determine whether the SWAXS profiles have strong correlations to the structural descriptors, we randomly generated structural descriptors within the corresponding domains and trained the random model. The performance of each model is reported in Fig. 3 ▸. In general, all the trained XGBoost models are stable and robust except for the *noisy* models of helical twist and major groove width. As expected, the random models all have significantly larger errors, which implies the existence of underlying patterns and a strong correlation between the SWAXS profile and structural descriptors of interest in this work. Fig. 4 ▸ shows the confusion matrices of all the trained XGBoost models applied to all training and testing sets.

Initially, we applied 10-fold cross validation (CV) using only 750 CARTs to quickly verify the statistical robustness of the model and to determine whether or not the XGBoost models suffer from overfitting. If the model is not robust, *i.e.* it might be sensitive to the order in which the training data were used or it can only be trained by a subset of training data, the validation performance among all 10 folds would have a large standard deviation. If the model overfits, the validation error would be large because the model does not generalize the learned pattern for an unknown dataset. We did not observe either phenomena except in the case of the *noisy* models of helical twist and major groove width. The 10-fold CV results are also reported in Fig. 3 ▸. It is also important to investigate how many SWAXS profiles (equivalent to the number of MD structures) are required to train the XGBoost model. Fig. S1 of the supporting information shows the mean squared error of 10-fold CV, training, validation and testing results versus the number of SWAXS profiles used to train the XGBoost model for a helical radius. The model error decreases monotonically as more profiles are used. In order for the trained model to generalize within an error tolerance of 0.01, about 15 000 profiles are required. The final XGBoost models are trained using an ensemble of 7500 CARTs (instead of 750 in 10-fold CV) with early stopping (Zhang & Yu, 2005 ▸; Yao *et al.*, 2007 ▸). The hyperparameters of our XGBoost models were the same and are reported in Table S1 of the supporting information. A snippet python script used to train XGBoost models to extract the helical radius and to reproduce some of the numbers/figures in this manuscript can be found at https://github.com/LP26/Pollack-Lab-Cornell/blob/master/WAXS-XGBoost-Radius-Training.ipynb.

We previously stated that the mapping between SWAXS profiles and structural descriptors is nonlinear, which justifies our choice of nonlinear ML models. To demonstrate this nonlinearity, we applied three linear models to the helical radius dataset: unregularized linear regression, ridge regression and least absolute shrinkage and selection operator (LASSO). The performance is reported in Table S2 and shows significantly large MSEs in training, validation and testing sets.

All the *noise-free*, *sparsely sampled* and *densely sampled* models were trained against the ‘over-sampled’ SWAXS profiles, containing more than 100 uniformly distributed *q* points. The Shannon sampling limit for our 12-base-paired RNA duplex system is 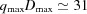. These profiles can successfully train the ML models, yielding consistent performance. It is worth investigating the behaviour of models trained by SWAXS profiles containing only the number of *q* points close to or below the Shannon sampling limit. Fig. S2 shows the performance of trained ML models on 10-fold CV, training, validation and testing sets versus the number of *q* points sampled for all structural descriptors. The panel at the bottom right shows the performance on random data (featureless data) as a control. The sampling limit is shown as a vertical line, to the left of which is the regime of ‘under-sampling’. As expected, under-sampling undermined the performance and stability of the XGBoost model, diminishing extraction of underlying structural information.

In addition to sampling effects, noise can also undermine the interpretation of SWAXS profiles. Our noisy models were trained with 5% noise (signal-to-noise ratio, S/N = 20) and demonstrate overfitting (as shown as the top two grey cells in Fig. 3 ▸). We explored how different signal-to-noise ratios affect our ML models. Fig. S3 shows the performance of trained ML models on 10-fold CV, training, validation and testing sets for all structural descriptors, versus different simulated noise levels, ranging from 7 to 30%. The panel at the bottom right shows the performance on random data as a comparison. This comparison shows that noisier data undermine the performance of the model, hiding structural information as increasing training MSE. Note that we used 750 CARTs in 10-fold CV and 7500 CARTs in training. However, the validation and testing traces fall close to the 10-fold CV trace when the noise exceeds 20% of the signal amplitude. This effect suggests that the ML models overfit the noise in the training data, performing poorly in both the validation and the testing sets. In other words, the ML models ‘learn’ the noise instead of the features which can be generalized for unknown SWAXS profiles. Therefore, to train ML models using a noisy input dataset, much more data must be included. These concerns are beyond the scope of this paper, which used simulated profiles (noise-free) from MD models.

### 3.2. Performance on synthesized noisy data   

**Figure 5:**
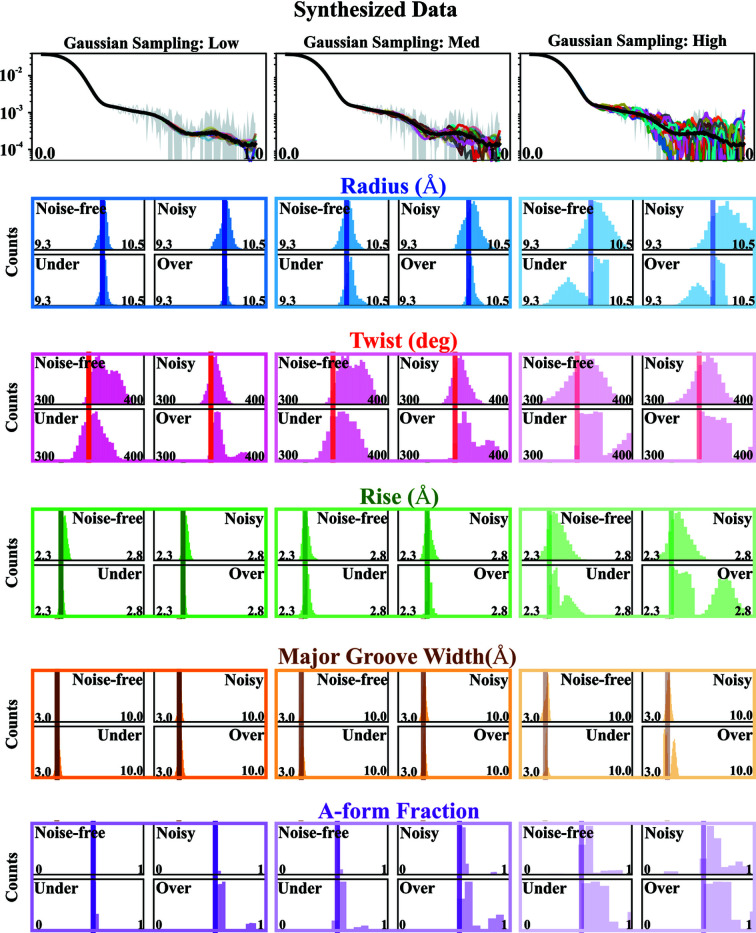
Performance of four trained XGBoost models on the noisy synthesized data from the testing set. Twenty sampled SWAXS profiles with low, medium and high error levels are shown in the top row. The subsequent rows show a number of boxed panels containing four histograms of predictions made by the different indicated models: *noise-free*, *noisy*, *sparsely sampled* and *densely sampled*. The vertical lines represent the real values, extracted from detailed molecular analysis. The transparency of the histograms is coded by the error levels: the higher the error, the more transparent the lines. Generally speaking, all the trained models perform well on noisy data with reasonable error levels (low and medium). As the error levels increase, corresponding to an unphysically low signal-to-noise ratio, outlier values start to appear, and the prediction distribution spreads. However, even under this extreme case, some of the peak values still recapitulate the real ones.

To investigate the effects of errors propagated by the trained ML models, we synthesized one noisy SWAXS profile from the testing set by catenating a third column of experimental error. We used this synthesized profile to compare the true values of structural descriptors with the outputs of trained ML models. The top panels of Fig. 5 ▸ show the synthesized data and errors along with a few (20) sampled curves with different error levels and corresponding predictions of the structural descriptors: helical radius (blue), twist (red), rise (green), major groove width (orange) and A-form fraction (purple) using four trained XGBoost models. The vertical lines represent the true values derived directly from the corresponding atomic conformation and the histograms show the distributions of the predictions. The transparency of the histograms denotes the error levels: the higher the error, the more transparent the histogram. In general, good performance is observed from trained XGBoost models: the peaks of the histograms recapitulate or are very close to the true values. As we increase our sampling error levels, larger variations are introduced in the output and the distributions spread out. However, the main peaks consistently recapitulate the real values until undermined or smeared out by other peaks incurred by noise. For noisy data, it is more robust to determine and interpret the most probable values of the structural descriptors rather than the mean of the distribution. Therefore, a trained XGBoost model can be applied to noisy data by Gaussian sampling with reasonable error levels.

### 3.3. Performance on experimental data   

**Figure 6:**
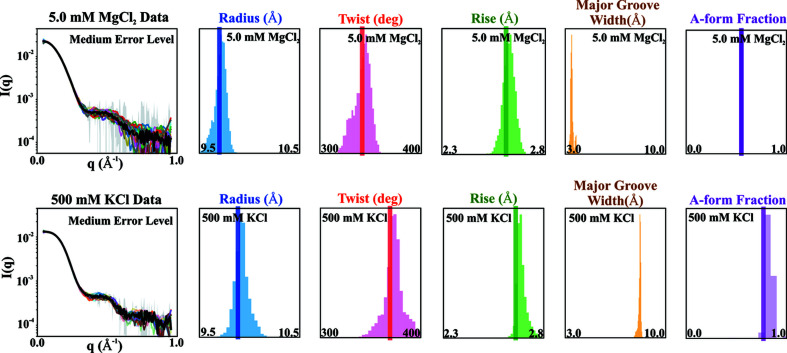
Performance of *noise-free* XGBoost models applied to experimental data acquired on dsRNA in 5.0 m*M* MgCl_2_ (top row) and 500 m*M* KCl (bottom row), respectively, using Gaussian sampling from medium-error levels. The real experimental values were obtained by curve-fitting using an extended ensemble optimization method. The major groove width was not reported in previous work, so its real value is missing. However, the predicted major groove width is about 3.5 and 7.5 Å for 5.0 m*M* MgCl_2_ and 500 m*M* KCl, respectively. For experimental data, the trained models still recapitulate the real values as means of prediction distributions.

Following the application of the ML approach to the synthesized profile, the same sampling and prediction procedures were applied to experimental data of the same 12-base-paired RNA duplex system in 500 m*M* KCl and 5.0 m*M* MgCl_2_. These conditions were selected based on recent results, showing significant differences in duplex conformation between the two salts. Previously, we established that divalent ions, like Mg^2+^, unwind and compress the dsRNA double helix, relative to the canonical A-form, distorting this geometry by reducing the major groove width to form a more compact conformation (Chen & Pollack, 2019 ▸). In contrast, at 500 m*M* KCl the duplexes are more relaxed and more conformationally similar to canonical A-form helices. Fig. 6 ▸ shows the predictions of XGBoost models from sampled experimental SWAXS profiles of duplexes in solutions containing 5.0 m*M* MgCl_2_ and 500 m*M* KCl using the medium error level and *noise-free* models. The ‘real’ values of the structural descriptors were obtained by full profile fitting and refinement of conformations described in the work by Chen & Pollack (2019 ▸) with the exception of the major groove widths, which were only inferred in the previous study. For comprehensive data visualization of different error levels and ML models, see Figs. S4 and S5 in the supporting information.

Good agreement is found between our ML approach and prior applied curve-fitting method. The dsRNA helical radius parameter has the strongest periodicity in this macromolecular system and XGBoost predicts this. Consistent with results from previous curve-fitting protocols, the helical twist has strong correlations with the major groove width because unwinding and over-winding of the duplex incur shrinkage and elongation of the major grooves, respectively, causing disruption or enhancement of helical periodicities. The helical rise is also well predicted by our trained XGBoost model. Although the length scale for helical rise is beyond the resolution of these measurements, it is reflected by the overall length of the duplex. Finally, the major groove exhibits strong helical periodicity because it is formed by repeated structuring of the phosphate backbones of the two hydrogen-bonded RNA single strands. The major groove width is 8.7 Å for the canonical A-form. However, the peak positions are about 3.5 and 7.5 Å for divalent and monovalent ions, respectively, suggesting the helical axial compaction is induced by divalent ions. The dsRNA adopts a more A-form-like conformation in the presence of monovalent ions; divalent ions compress it with significant deviation from the canonical A-form duplex. Comparison of these results with those from a curve-fitting method suggests that XGBoost models demonstrate the same conformational trends.

### 3.4. Interpretation of XGBoost models   

The molecular radius of gyration is obtained from intensities in the lowest *q* region using a small-angle approximation of the Debye formula. Beyond this lowest *q* region, different molecular shapes have different 1D features. Some important conformational information can be assessed from higher angle data, for example through Kratky plots where compaction of a molecular system is reflected by a strong peak, whereas an unfolded random coil has a different shape. However, in general, more detailed structural information is hidden in the 1D profile and is difficult to extract without knowledge of the system or theoretical molecular models.

**Figure 7:**
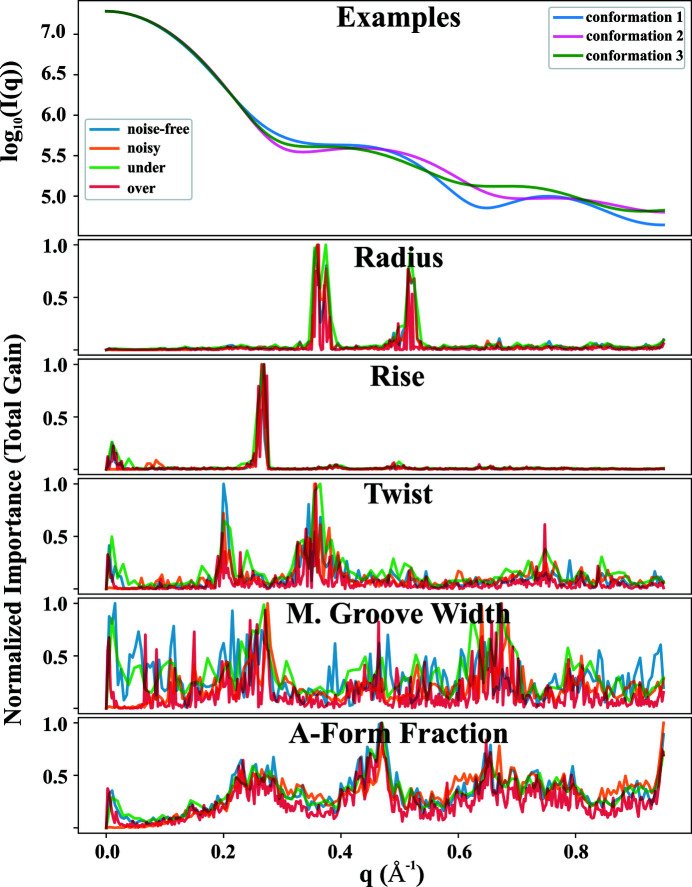
Normalized ‘gain-importance’ traces for four trained models. The ‘gain-importance’ reports the significance of the scattering intensities in predicting a certain structural descriptor. Intensities at different locations along the *q* axis have different significance, suggesting that the information content is not uniformly distributed in *q*. A more detailed description is provided in the text.

Our trained XGBoost model has the ability to correlate features in the profiles to real-space structural descriptors. The ‘importance value’ of each feature provides essential insight into decoding the 1D scattering profile. Fig. 7 ▸ shows the normalized ‘gain importance’ of the trained XGBoost models on different structural descriptors. The gain importance reports a prediction power of scattering angles. Among all four trained XGBoost models (*noise-free*, *noisy*, *sparsely sampled* and *densely sampled*), the gain-importance traces are consistent, suggesting that the models extract and detect the same underlying features regardless of the sampling or noise in the training data. Interestingly, for some structural descriptors, certain regions along the *q* axis are more critical in making predictions. For example, scattering intensities in the region near *q* = 0.30 and 0.55 Å^−1^ appear to be critical for helical radius prediction. This correlation is intuitive, because these *q* regions correspond to real-space dimensions of the duplex diameters and radii, which are well represented in these structures. On the other hand, counter-intuitively, intensities near the relatively low *q* region *q* ≃ 0.25 Å^−1^ are of high significance in predicting the structural descriptor of the smallest length scale: the helical rise. However, the helical rise can be cast as the average di-base-pair distance in the axial direction, and therefore reflect the total length of the RNA duplex. In this work, the 12-bp duplex ranges from 25 to 30 Å in length, consistent with the appearance of a signature near *q* ≃ 0.25 Å^−1^. Moreover, from Fig. 7 ▸, the helical twist is reflected near *q* ≃ 0.25 and 0.35 Å^−1^ in the SWAXS profiles while the major groove width is correlated to the second local extremum. The A-form fraction summarizes of all these helical structural determinants and is mostly predicted by combining all the SWAXS features; it relies on features present over the full angle range sampled.

It is also interesting to understand and visualize how the XGBoost model makes predictions. This decision-making process is evident by considering the other type of importance: ‘weight-importance’, which reports the number of times a feature is used by the model in the decision rules. The normalized ‘weight-importance’ traces are shown in Fig. S6. All of the traces are very similar regardless of structural descriptors or XGBoost models, suggesting that the prediction is made through almost identical processes distinguished by the gain, shown in Fig. 7 ▸.

The ML model mines those hidden patterns and structural information. The structural information is not distributed uniformly across the scattering angles, but perhaps not surprisingly, appears at or near *q* values that report on a particular length scale.

### 3.5. Final remarks   

Over the past few years, increasing efforts have been made to bridge MD simulations with experimental data to gain understanding of macromolecular systems and hence biological insight. The application of ML opens the door to a new perspective. To help guide future explorations and based on the work described above, we briefly discuss some potential limitations of this approach and, where we can, provide suggestions that may benefit future studies. First, the conformational sampling of MD might undermine any structural interpretation based on features found in the scattering profiles. Researchers are advised to check the diversity of conformations found in the simulations. For example, we ensured that the experimental data in the full SWAXS regime is bound by scattering profiles computed from conformations in the MD simulations. One must also be aware of limitations caused by the inaccuracies of force fields or other sampling issues. However, even with well sampled MD models, perfect agreement between a certain model and experimental data is not expected. ML can help by identifying or ‘learning’ which features of the data reflect specific aspects of the structure. Second, the structural descriptor of interest must be ‘detectable’ in the simulated data. Its presence can be verified using the training set to allow the model to recognize a descriptor, then verifying the recognition. It is important to ensure that significant patterns, visible to humans or not, are accurately mapped by this structural descriptor. Third, overfitting the data is always a concern. We recommend that researchers perform validation tests on the ML models, especially those trained by noisy data. Moreover, the trained ML models are specific to one type of macromolecule (*e.g.* 12-base-pair duplex), unless a training set that incorporates a diverse set of systems is employed. Importantly, this analysis pipeline can be readily extended to different molecular systems by bridging MD simulations and experimental data. Finally, we would like to emphasize the benefits of ‘learning from’ the ML interpretations. In the best case, researchers build intuition based on feature identification from the trained ML models, *e.g.* the feature at this *q* value contains information about the helical radius. We envision future applications where both people and machines ‘learn’ from the data to increase our understanding of biological macromolecules.

## 4. Conclusions   

Our goal is to propose ML frameworks for the analysis of solution X-ray scattering data when MD predictions are available. To the best of our knowledge, this is a novel approach to model scattering over a full spectrum of angles; importantly, this includes wide angles that provide information about smaller length scales.

This work presents an ML framework based on extreme gradient boosting (XGBoost) that bridges models from molecular dynamics simulations with experimental solution X-ray scattering measurements. Taken together, this approach provides important structural descriptors for regular macromolecular motifs, such as the important dsRNA double helical structure. Our models accurately predict helical radius, twist, rise, major groove width and A-form fraction. Trained ML models were applied to experimental SWAXS data of the same system in different salt-containing solutions, 500 m*M* KCl and 5.0 m*M* MgCl_2_, where helical conformational changes are known to vary. Previously published results have been reproduced, and the ML models confirm that divalent ions unwind and shrink the dsRNA duplex, rendering a tighter major groove and further deviation from canonical A-form geometry.

Improved performance of XGBoost models may be achieved by further variation of hyperparameters, training strategies and even differing data representation [for example, changing from log*I*(*q*) − *q* to log*I*(*q*) − *q* ^2^]. Better performance may be realized by complex ML models such as neural networks. This ML methodology can be applied to a wide range of molecular systems to derive structural parameters of interest with high confidence to understand conformational changes and structures encoded in SWAXS profiles. We envision potential applications of similar or improved frameworks in different molecular systems, data acquired by other experimental techniques or analyses that do not require predetermination of structural parameters. In such context, this work can be viewed as a case study for applying ML algorithms to bridge theoretical models with experimental work on complex yet essential molecular systems.

## 5. Related literature   

The following references are cited in the supporting information: Macke & Case (1998 ▸); Phillips *et al.* (2005 ▸); Huang *et al.* (2017 ▸); Jorgensen *et al.* (1983 ▸); Humphrey *et al.* (1996 ▸); Essmann *et al.* (1995 ▸).

## Supplementary Material

Additional figures. DOI: 10.1107/S2052252520008830/yu5020sup1.pdf
