# Supplementary material for: Machine learning deciphers structural features of RNA duplexes measured with solution X-ray scattering
Source: IUCrJ. 2020 Aug 12;7(Pt 5):870–80. doi: 10.1107/S2052252520008830 (PMC7467162; doi:10.1107/S2052252520008830)

IUCrJ

Volume 7 (2020)

Supporting information for article:

Machine Learning Deciphers Structural Features of RNA Duplexes Measured with Solution X-ray Scattering

Yen-Lin Chen and Lois Pollack

S1. Hyperparameters of the XGBoost Model

We used the same set of hyperparameters to train all of our XGBoost models. Parameters did not require extensive tuning because the SWAXS features are strongly correlated to the structural descriptors. The hyperparameters are reported in Table S1.

Table S1 The hyperparameters of the XGBoost model.

Hyperparameter name	XGBoost Arguments	Value
Learning Rate	`learning_rate`	0.07
Maximum Tree Depth	`max_depth`	3
Number of Trees	`n_estimators`	750 (10-fold cross-validation)<br>7,500 (Training w/ early stopping)
L1 regularization term	`reg_alpha`	0.75
L2 regularization term	`reg_lambda`	0.45
Subsample ratio of columns	`colsample_bytree`	0.4
Subsample ratio	`subsample`	0.8
Minimum sum of instance weight	`min_child_weight`	1.5

S2. Performance of Linear Models

We benchmarked the XGBoost models with unregularized linear models, Ridge regression and least absolute shrinkage and selection operator (LASSO). The Ridge regression and LASSO correspond to L2 and L1 regularization of the linear model. We trained the linear models using the helical radius data. The mean-square-error (MSE) is reported in Table S2. The performance of these linear models is not comparable to that of XGBoost model based on the large MSE. This comparison implies the nonlinearity of SWAXS profiles and helical radii of the RNA duplexes.

Table S2 Performance of linear models using the helical radius dataset. The result should be compared to the *noise-free* XGBoost model in Table 1 in the main text. The regularization coefficient is α.

	Unregularized Linear Model	Ridge Regression $\alpha = 0.05$	LASSO $\alpha = 0.2$
Training MSE	0.013	0.017	0.067
Validation MSE	0.013	0.017	0.069
Testing MSE	0.014	0.018	0.070

S3. Setup of MD Simulations

The setup for the MD simulations was described in section A.1 of (Templeton & Elber, 2018), where a helix-junction-helix (HJH) conformation was studied. The simulation parameters are paraphrased below.

The helix HJH construct of interest was composed of two 12 base pair A-form RNA duplexes, connected by a linker, consisting of a poly(U) junction with five nucleotides. One long strand, with sequence 5'-CCCUAUACUCCCUUUUUCCUCCUAAUCGC-3' was base paired at each end (for 12 residues) with a complementary strand to form the HJH complex. Initially, the construct was created using the make-na web server[1], an online platform that builds single or double stranded helical nucleic acids. The MD simulations were performed using NAMD[2] as well as the CHARMM 36 force fields[3].

Water molecules from TIP3P [4] solvated the molecules within a 100x100x120 $Å^3$ periodic box. Ions were randomly placed using the VMD autoionize plugin[5]. Ions were initially located at least 5 Å from the RNA or other ions. Temperature was maintained at 310K, and pressure was 1 atmosphere. Langevin dynamics and a Nose-Hoover Langevin piston were used for all simulations. For electrostatics, Particle Mesh Ewald (PME) summation[6] with grid spacing of 1 Å was used. A 12 Å cutoff distance was used to evaluate Lennard-Jones forces. One helix was consistently kept rigid to fix the RNA and to provide a static frame of reference. The other was allowed to fluctuate, and it is the latter helix that was used for comparisons in this work.

A free energy landscape was generated using Milestoning with radius of gyration as variable. Simulations were conducted at 30 mM $MgCl_2$ and at 60 mM KCl.

[1] Macke, T. J.; Case, D. A., Modeling Unusual Nucleic Acids, in *Molecular Modeling of Nucleic Acids* **1998**, 682, 379.

[2] Phillips, J. C.; Braun, R.; Wang, W.; Gumbart, J.; Tajkhorshid, E.; Villa, E.; Chipot, C.; Skeel, R. D.; Kale, L.; Schulten, K. Scalable Molecular Dynamics with NAMD *J. Comput. Chem.* **2005**, 26, 1781.

[3] Huang, J.; Rauscher, S.; Nawrocki, G.; Ran, T.; Feig, M.; de Groot, B. L.; Grubmuller, H.; MacKerell, A. D. CAHRMM36: An Improved Force Field for Folded and Intrinsically Disordered Proteins *Biophys. J.* **2017**, 112, 175A

[4] Jorgensen, W. L.; Chandrasekhar, J.; Madura, J. D.; Impey, R. W.; Klein, M. L. Comparison of Simple Potential Functions for Simulating Liquid Water, *J. Chem. Phys*. **1983**, 79, 926.

[5] Humphrey, W.; Dalke, A.; Schulten, K. VMD: Visual Molecular Dynamics *Journal of Molecular Graphics & Modelling* **1996**, 14, 33.

[6] Essmann, U.; Perera, L.; Berkowitz, M. L.; Darden, T.; Lee, H.; Pedersen, L. G. A Smooth Particle Mesh Ewald Method *J. Chem. Phys.* **1995**, 103, 8577.

Figure S1 The mean squared error (MSE) of the 10-fold CV, training, validation and testing results versus the number of MD structures used to train the XGBoost model for helical radius. To achieve a higher accuracy in the trained XGBoost model on the testing set (higher power of prediction and generalization), a larger dataset is required for training. For error tolerance of 0.01 in the helical radius parameter for the 12-base-paired duplex, at least 15,000 MD structures are required.

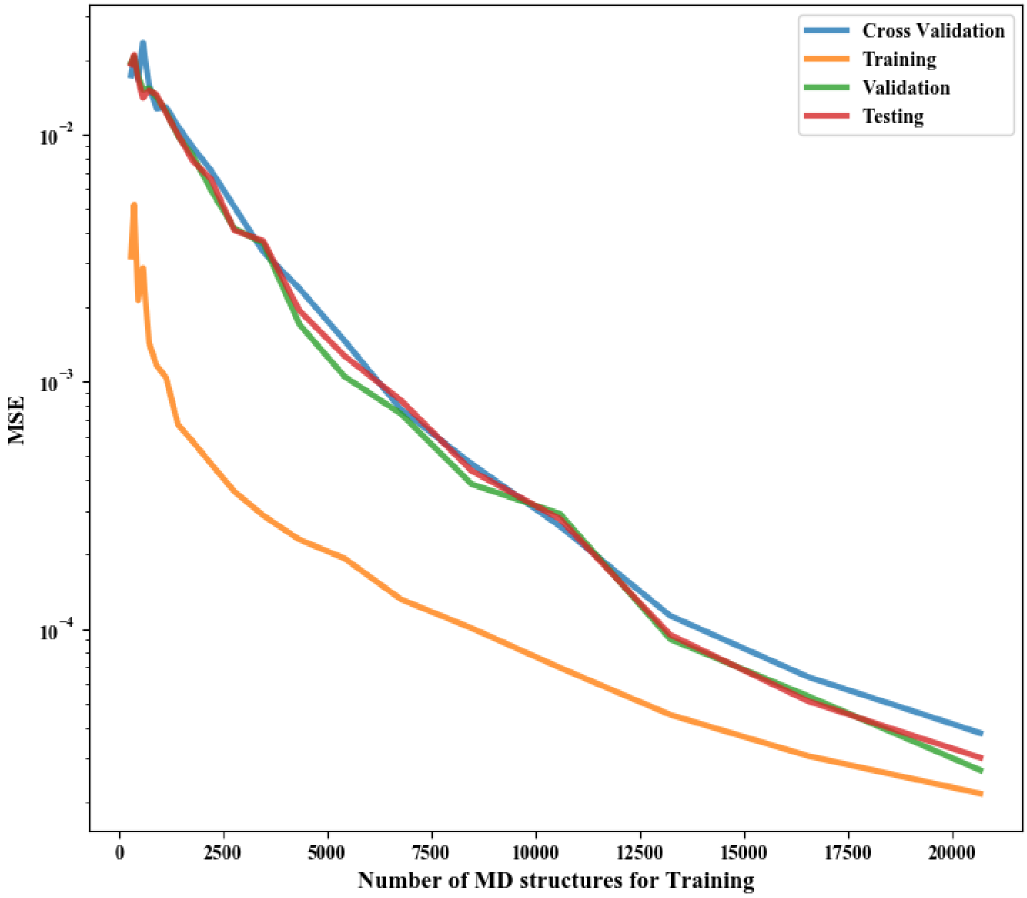

Figure S2 The performance of XGBoost models trained by SWAXS profiles with different numbers of q points close to or under the Shannon sampling limit for all the structural descriptors and random data as a control. The vertical line is the sampling limit, about 31 q points for our 12-based-paired system. In the under-sampled regime, the performance rapidly degrades, losing the structural information in the SWAXS profile and barely trains the model.

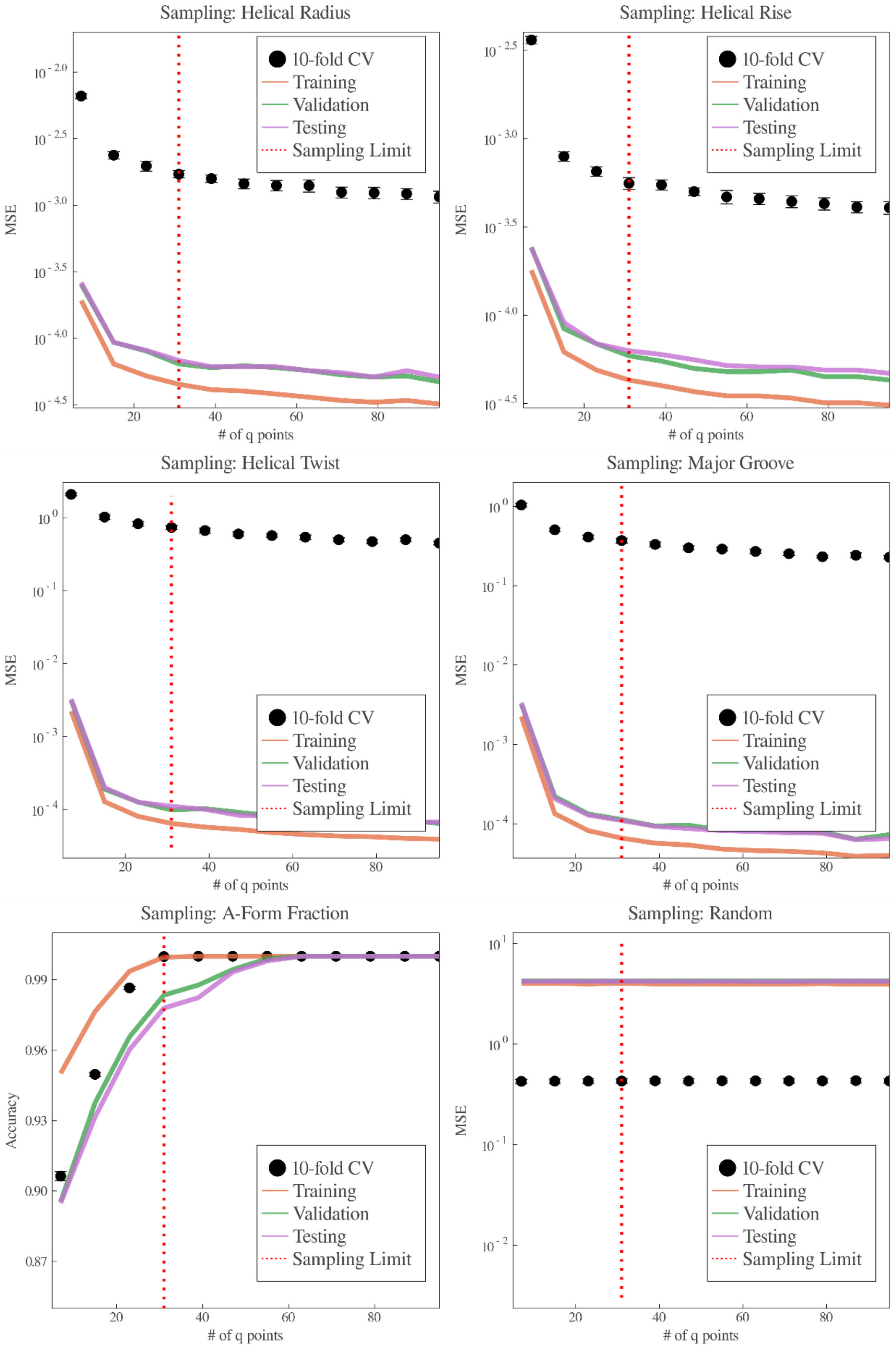

Figure S3 The performance of XGBoost models trained by noisy SWAXS profiles with noise percentages ranging from 5% to 30% for all the structural descriptors and random data as a control. The signal-to-noise ratio is the reciprocal of the noise percentage. As the noise increases, the structural information is less clear in the SWAXS profile, so the ML models perform poorly as training error increases. The inclusion of noise in the training data increases the risk of overfitting since ML models are likely to learn from noise, resulting in greater errors in the validation and testing sets.

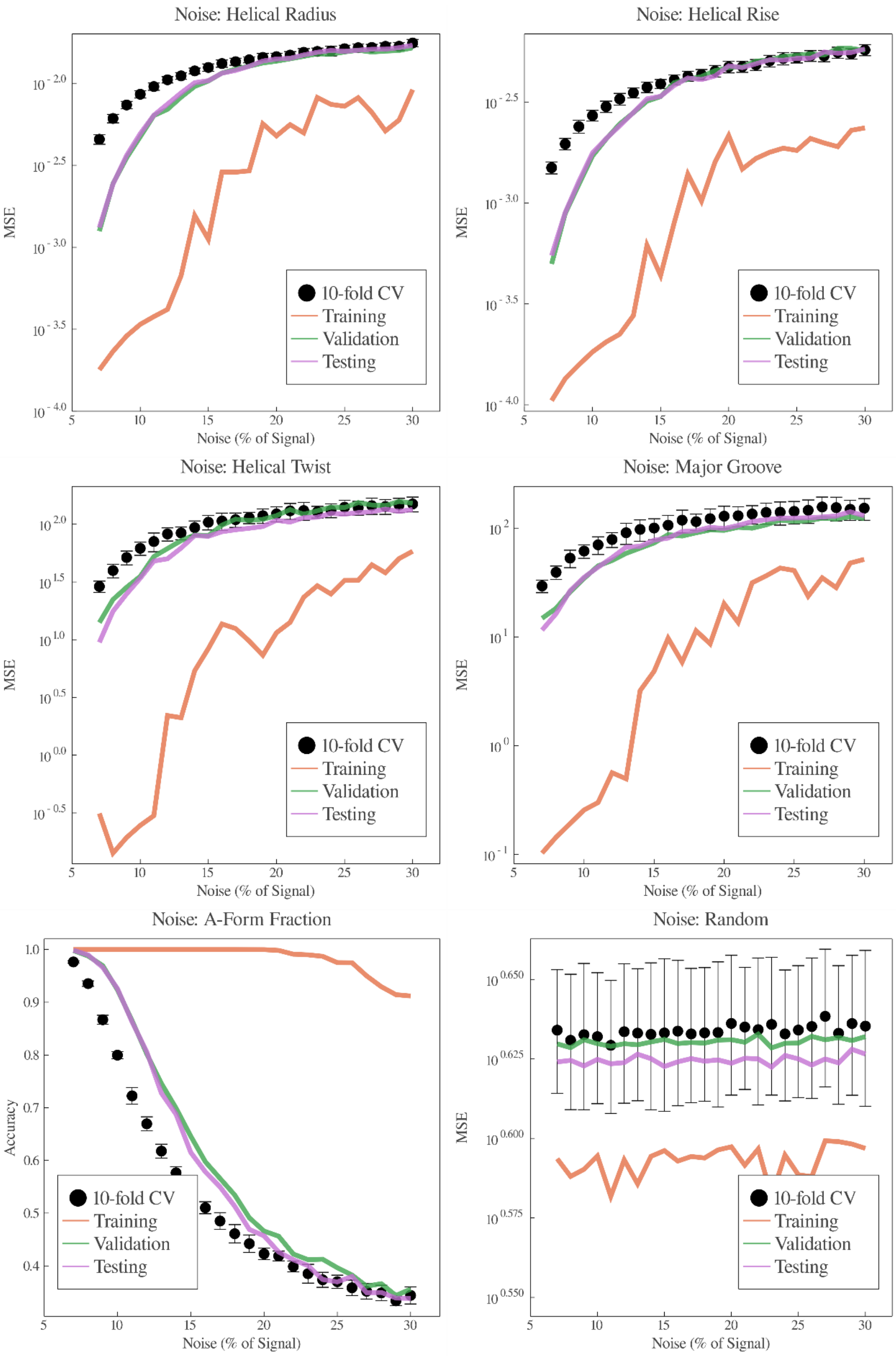

Figure S4 The performance of 4 trained XGBoost models on the experimental data of the 12 bp RNA duplex in a solution containing 5.0 mM $MgCl_2$. The visualization scheme is as described in Fig. 4 in the main text.

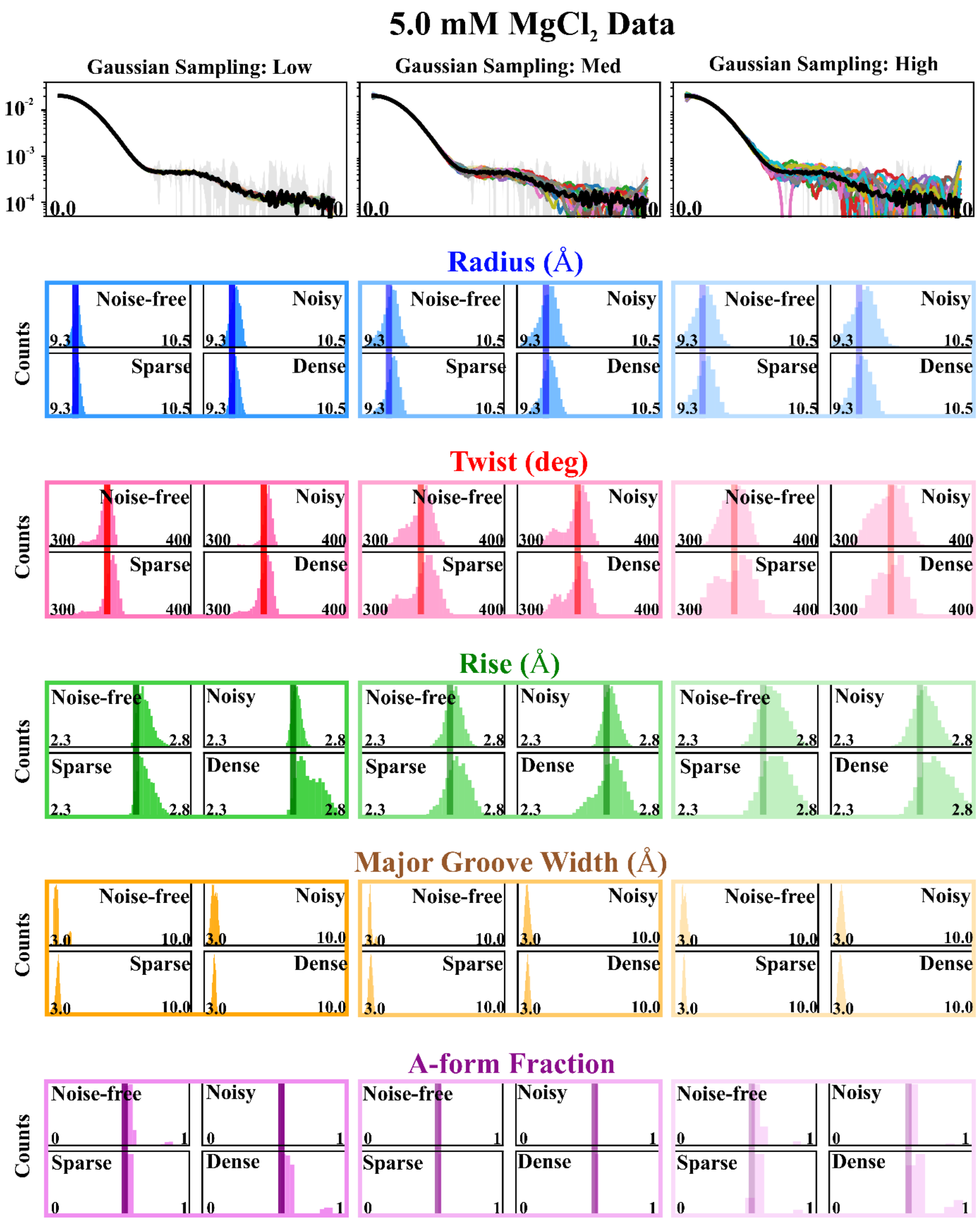

Figure S5 The performance of 4 trained XGBoost models on the experimental data of the 12 bp RNA duplex in a solution containing 500 mM KCl. The visualization scheme is as described in Fig. 4 in the main text.

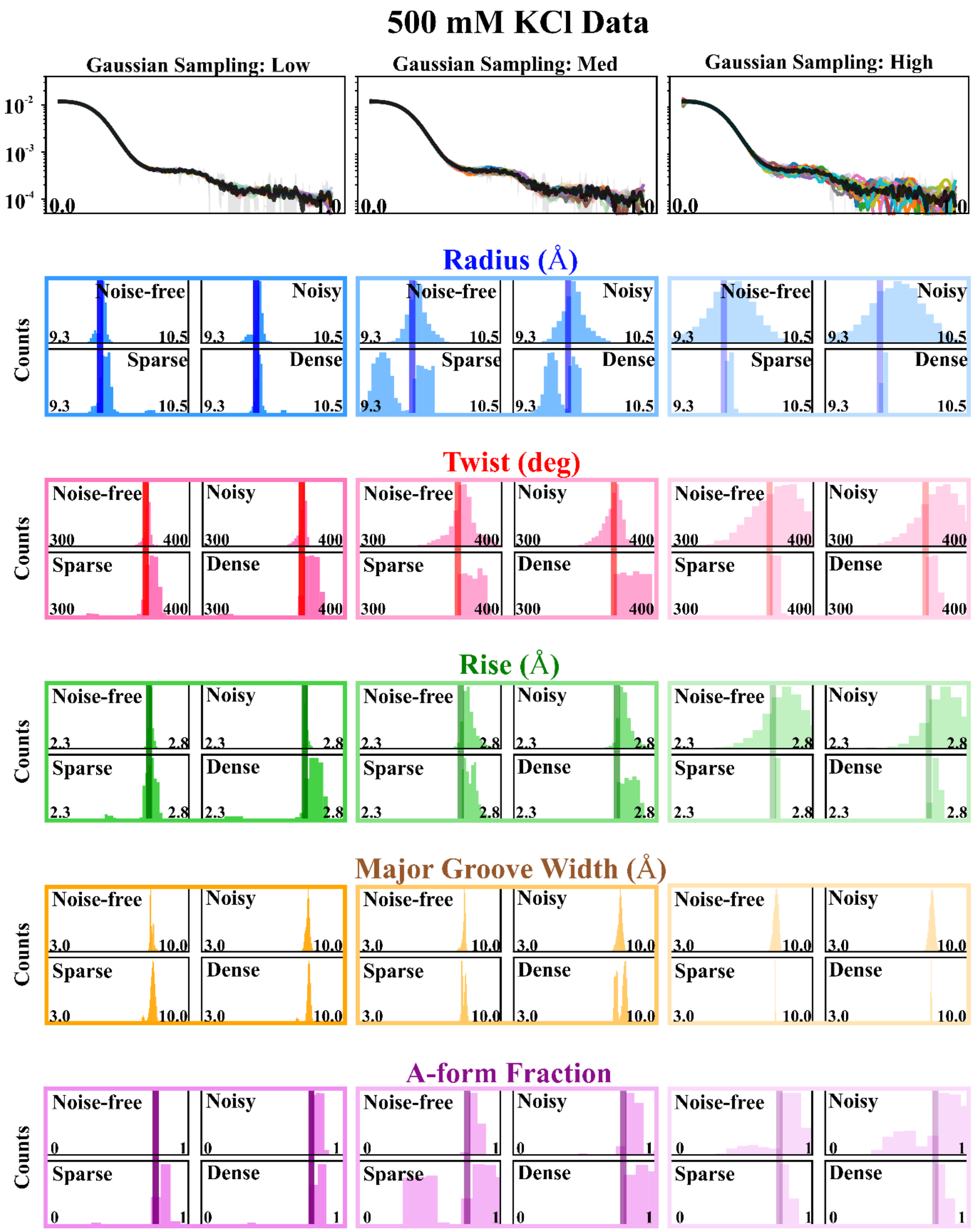

Figure S6 The normalized "importance-weight" traces for 4 trained XGBoost models. The "importance-weight" reports the number of times a feature (here the intensity at certain q) is used in the model to make predictions. It reflects the decision-making process of the model. Among all the trained models and structural descriptors, the traces are very similar, suggesting almost identical splitting of the CARTs in the tree boosting ensemble. The difference lies only in the "gain" associated with each intensity, reported in Fig. 6 in the main text.

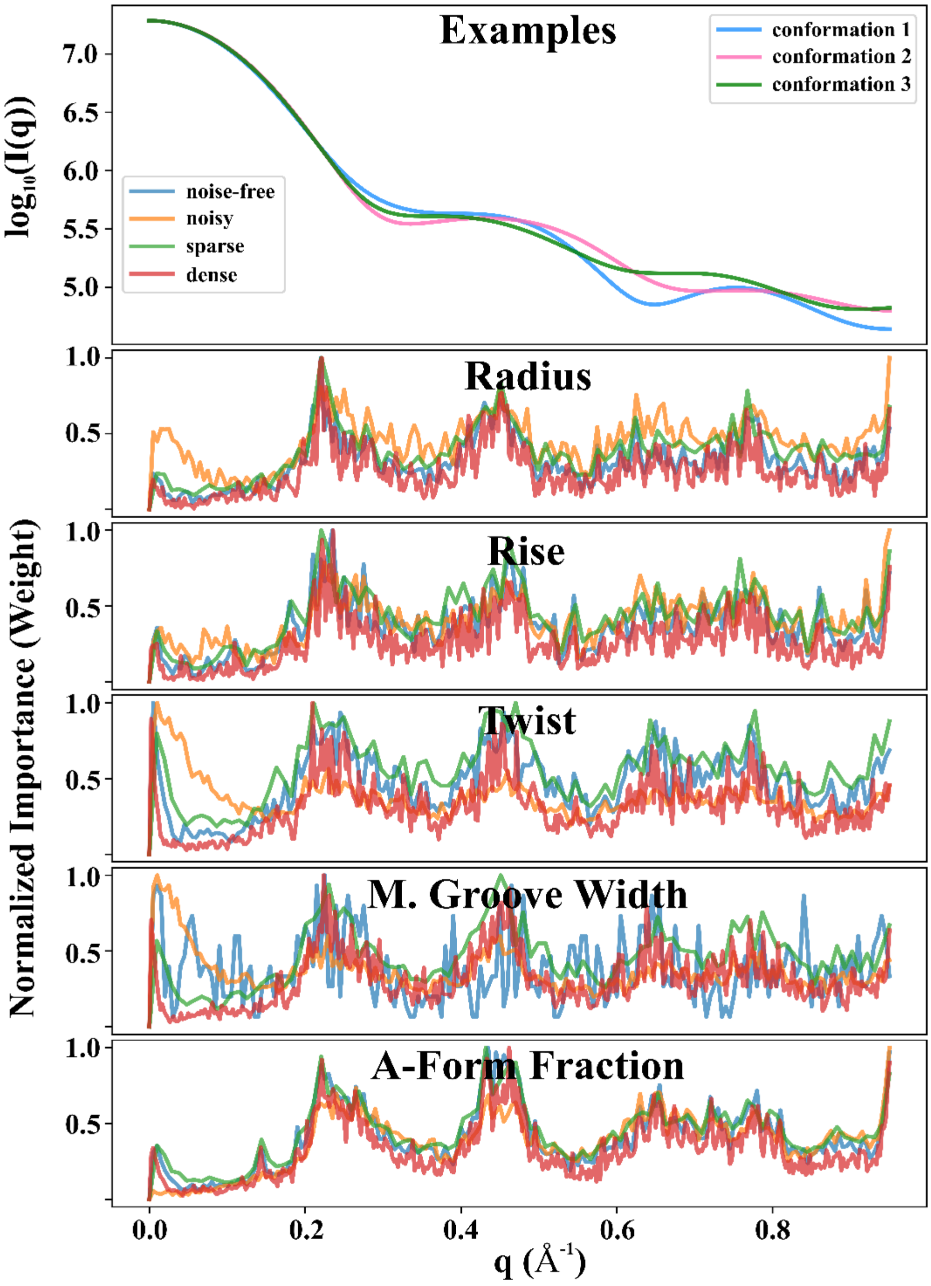

Supplement: Supplementary file 1 [file m-07-00870-sup1.pdf]
